# Molecular Mechanisms of Glial Cells Related Signaling Pathways Involved in the Neuroinflammatory Response of Depression

**DOI:** 10.1155/2020/3497920

**Published:** 2020-10-10

**Authors:** Junhui Wang, Jie Qin, Peng Wang, Yu Sun, Qi Zhang

**Affiliations:** ^1^The Lunenfeld-Tanenbaum Research Institute, Mount Sinai Hospital, Toronto, Ontario, Canada; ^2^Department of Ultrasound, Qingdao Municipal Hospital, Qingdao, Shandong, China; ^3^School of Basic Medicine, Qingdao University Medical Center, Qingdao, Shandong, China; ^4^Department of Endocrinology, Qilu Hospital of Shandong University, Jinan, Shandong, China; ^5^Department of Pathology and Laboratory Medicine, Schulich School of Medicine and Dentistry, Western University, London, Ontario, Canada

## Abstract

Dysfunction of the glial cells, such as astrocytes and microglia, is one of the pathological features in many psychiatric disorders, including depression, which emphasizes that glial cells driving neuroinflammation is not only an important pathological change in depression but also a potential therapeutic target. In this review, we summarized a recent update about several signaling pathways in which glial cells may play their roles in depression through neuroinflammatory reactions. We focused on the basic knowledge of these signaling pathways by elaborating each of them. This review may provide an updated image about the recent advances on these signaling pathways that are essential parts of neuroinflammation involved in depression.

## 1. Introduction

Depression is one of the most common disability diseases in human beings, affecting approximately 16% of the world's population. In addition to major depressive disorder (MDD) patients, there are many diseases associated with depression, such as Alzheimer's disease, epilepsy, cerebrovascular disease, cancer, and Parkinson's disease [1–3]. So far, the mechanisms of depression are understood to be serotonin deficits, stress, and the hypothalamic-pituitary-adrenal axis theory [4–6]. In recent years, the theory of inflammation is attracting much more attention. Clinical and basic studies have revealed that immunological abnormalities and cytokines could significantly affect depressive symptoms, the neuroendocrine system, neurotransmitters, neurodegeneration, and neurogenesis [7–9].

Microglia and astrocytes are important immune regulatory cells in the central nervous system (CNS). Usually, microglia is named as “resting” cells, with long branching processes and a small cellular body, which keep monitoring immune threats while maintaining homeostasis in the CNS [10]. When activated, ramified microglia could turn into ameboid morphology, phagocyte-like cell, and secrete immune-molecules, including a series of cytokines [10]. During this course, the activated microglial cells could be transformed to either MI type (toxic) or M2 type (protective) microglia, depending on the variety of external or internal insults [11]. Astrocytes, key components of the blood-brain barrier (BBB), serve as a functional barrier that regulates and restricts CNS inflammation [12]. In addition, astrocytes could regulate and keep the balance of the glutamate system and significantly contribute to synaptic plasticity [13].

Patients with MDD showed elevated microglial density in the frontal cortex, temporal cortex, and hippocampus from a positron emission tomography (PET) study [14]. In the same study, the expressions of some genes in astrocytes were decreased in the prefrontal cortex of MDD patients, which reflected an astrocytic dysfunction, such as GFAP, ALDH1L1, SOX9, GLUL, SCL1A3, GJA1, and GJB6 [15]. The findings from human beings are consistent with results from animal models of depression [16]. Neuroinflammation with increased expression of proinflammatory cytokines in CNS contributes to the etiology of depression. Animals with proinflammatory cytokines injection exhibit depression-like behavior [17]. Intraperitoneal injection of lipopolysaccharide (LPS) that could trigger systemic inflammation led mice to show obvious depressive-like behaviors, with activated microglia and astrocytes in the hippocampus and medial prefrontal cortex (mPFC) [17]. Previous reports also found that rats under the chronic unpredictable mild stress (CUMS) model exerted depressive-like behavior with obvious hippocampus and mPFC microglia activation and reactive astrogliosis [18, 19]. After maternal deprivation, the level of glial fibrillary acidic protein (GFAP) immunopositive cells decreased in the hippocampus of offspring rats in early developmental phases but increased in late developmental phases [20]. Iba-1 (microglial marker) immunopositive cells increased in both early and late developmental phases [20]. These data suggested that microglia and astrocytes might play key roles in MDD. Maternal deprivation is a well-established predisposing factor for the development of anxiety and depression [21].

Therefore, microglia and astrocytes could be extensively involved in the pathogenesis of both MDD patients and animal models. The underlying mechanisms by which these glial cells trigger the neuroinflammatory response, and how they are orchestrated and regulated by each other, are quite important in order to understand the significance of these events of glial cells during the development of depression. Therefore, we summarized the recent research findings of this field and presented the updated profile of the network of glial cells, neuroinflammation, and depression.

## 2. Signaling Pathways of Neuroinflammation in Depression

### 2.1. Kynurenine Pathway

The kynurenine pathway is regulated by inflammatory cytokines in CNS diseases [22]. Tryptophan (TRP) is transformed to kynurenine (KYN) by tryptophan dioxygenase or indoleamine 2, 3-dioxygenase (IDO). With the help of kynurenine aminotransferase (KAT), KYN is transformed into kynurenic acid (KYNA), which is an antagonist of N-methyl-D-aspartic acid (NMDA) receptor and *α*7-nicotinic acetylcholine receptor [23]. KYN can also be transformed into 3-hydroxykynurenine (3-HK) with the kynurenine monooxygenase (KMO). Then, 3-HK is transformed to quinolinic acid (QUIN), which is an agonist of the NMDA receptor [24]. Since literatures have shown kynurenine signaling could significantly impact glutamate, acetylcholine, and serotonin pathways, the above KYN pathways of tryptophan are considered to play important roles in the pathophysiology of inflammation and depression [25]. A study showed that IDO and KMO were significantly activated to produce large amounts of 3-HK and QUIN in microglial cells exposed to IFN-*γ* stimulation [26]. Combination treatment of IFN-*γ* and LPS in microglial cells leads to significant IDO upregulation, which was not inhibited by nitric oxide (NO) [27]. In another study with an animal model, results suggested that LPS injection induced the microglial and IDO activation, and the activation could be exacerbated in CX3CR1 (-/-) deficient mice, which implied that CX3CR1 could inhibit the activation of IDO [28]. A previous report also validated that suppression of the KYN pathway in microglia could significantly reduce the neurite branching and complexity of cortical neurons [29]. By combination with L-KYN and LPS, L-KYN exerted a significant inhibitory effect on the microglia response to LPS, which suggested that the KYN pathway might play a direct role in regulating microglia activity [30].

While astrocytes have limited expression profiles of the above enzymes, IDO was activated by IFN-*γ* in astrocytes, which only resulted in large amounts of KYN and KYNA, but not the generation of 3-HK and QUIN [26, 31]. However, KYN and KYNA in astrocytes could be released and absorbed by surrounding microglia, which would further promote the neuroinflammatory response. LPS increased the expression of IDO-11-FL in both astrocytes and microglia in the mice brains, but IDO-2-v6 was only induced in astrocytes [32]. The different profiles of this signaling pathway in astrocytes and microglia should be taken into account in future studies.

### 2.2. Inflammasome

The activation of the inflammasome participates in the innate immune reaction in depression [33]. The NOD-like receptor (NLR) family pyrin domain containing 3 (NLRP3) inflammasome, which includes the NLRP3 protein, adapter protein apoptosis-associated speck-like protein (ASC), and procaspase-1, is the most studied member of the inflammasome [34]. In an *in vitro* study, primary human microglial cells contained the expression profile of inflammasome-related genes and the expression of these genes could be regulated functionally [35]. LPS-induced activation of NLRP3 and caspase-1 could be detected in microglia, and this may be correlated to microglia polarization to the M1 phenotype [36]. In primary cultures of astrocytes, oxygen-glucose deprivation and reoxygenation could induce the upregulation of NLRP3, caspase-1, and the extracellular release of IL-1*β* and high mobility group box 1 (HMGB1) [37]. Furthermore, the NLRP3 inflammasome complex could be expressed in astrocytes induced by HMGB1, and IL-4 could inhibit this effect through a negative regulation of NF-*κ*B activity and promotion of peroxisome proliferator-activated receptor *γ* (PPAR*γ*) activation [38]. In MDD patients and depressive rodents, assembly of the NLRP3 complex, the subsequent proteolysis, and release of the proinflammatory cytokines interleukin-1*β* (IL-1*β*) and IL-18 have been widely reported [39]. LPS-injected mice displayed elevated expressions of NLRP3, ASC, and caspase-1 in the hippocampus, and this was correlated with long-term depression-like behaviors [40]. In the CUMS rat model, NLRP3 inflammasome was activated in microglial cells of the PFC area, and subsequently, IL-1*β* was elevated as well [41].

### 2.3. Purinergic Pathway

Purinergic receptors are divided into ionotropic P2X (for ATP) and metabotropic P1 (for adenosine) or P2Y. The P2X7 receptor (P2X7R) is increasingly recognized as an important cell surface regulator of some key inflammatory molecules, including IL-1*β*, IL-18, IL-6, and tumor necrosis factor-alpha (TNF-*α*). Moreover, a study has proven that the generation of P2X7R-dependent cytokines is driven by the activation of NLRP3 inflammasome and antagonists of P2X7R, which is likely to possess therapeutic potential as novel anti-inflammatory therapies [42]. In depressive mouse models induced by LPS or CUMS, the P2X7/NF-*κ*B pathway was activated in the hippocampus or mPFC of the mouse brain, and the expressions of p-IKK*α*, p-IKK*β*, p-I*κ*B*α*, p-NF-*κ*B, p65, IL-6, IL-1*β*, and TNF-*α* were elevated as well [43, 44]. The activation of P2X7R was accompanied by the inflow of Ca^2+^, followed by the activation of MAPK kinases (ERK, JNK, p38), transcriptional factors (NF-*κ*B, CREB, AP-1) into the nucleus, and the boosted expression of series of inflammatory genes (TNF-*α*, IL-6, COX-2, iNOS, IL-2, IFN-*γ*, IL-3, etc.). Likewise, the activation of P2Y1 receptor (Gq-coupled proteins) could lead to the activation of PLC and subsequently mobilize intracellular calcium, which could cause the activation of PKC, p38 MAPK, and transcriptional factor CREB, thus regulating the expression of inflammatory genes [45].

In cultured microglia, the release of IL-6, IL1*α*, IL1*β*, IL18, and chemokine CC motif ligand 2 (CCL2) was P2X7R-dependent, and benzoyl-benzoyl ATP (Bz-ATP) could also induce microglial cell death [46, 47]. IL-1*β* release was P2X7R-pore-dependent, and IL-1*β* had trophic effects on surrounding microglia in terms of their activation and proliferation [48]. In astrocyte cultures, ATP increased Ser-727 phosphorylation of signal transducer and activator of transcription 3 (STAT3), which played an important role in astrocyte proliferation and reactive astrogliosis [49]. Selective P2X7R agonist, Bz-ATP, increased monocyte chemoattractant protein-1 (MCP-1) expression through the activation of ERK1/2 and p38, which would make leukocyte infiltration in the CNS after inflammation [50].

### 2.4. Nicotinic Acetylcholine Pathway

In depression, central alpha7 nicotinic acetylcholine receptor (*α*7 nAChR) is a key player in regulating the cholinergic mediated anti-inflammatory pathway [51]. The *α*7 nAChR positive allosteric modulator (PAM), PNU120596, prevented LPS-induced depression-like behaviors in mice, hindered activation of microglia and astrocytes, and inhibited the upregulation of IL-1*β* and TNF-*α* in the hippocampus and prefrontal cortex [52]. The *α*7 nAChR-signaling pathway was involved in the process of nicotine regulation of microglia activation with a neuroprotective role. In cultured microglia, nicotine suppressed LPS-induced TNF-*α* release via the activation of *α*7 nAChRs, a signaling process involved the activation of PLC and Ca^2+^ release from intracellular Ca^2+^ stores, and associated with the suppression of ERK, JNK, and p38 activation [53]. Meanwhile, nicotine enhanced P2X7-receptor-mediated TNF-*α* release from microglia [53]. The activation of *α*7 nAChRs by nicotine caused the upregulated expression of cyclooxygenase-2 (COX-2) and prostaglandin E2 (PGE2) in microglial cultures [54] and elevated the expression of glutamate/aspartate transporter (GLAST) and glutamate uptake [55].

The expression of *α*7nAChR was detected in human astrocytes as well. In cultured astrocytes, pretreatment with nicotine could suppress MPP or LPS-induced TNF-*α* expression and activation of ERK1/2 and p38 [56]. After stimulation by IL-1*β*, nicotine could inhibit the proinflammatory cytokines, such as IL-6, IL-1*β*, TNF-*α*, IL-8, and IL-13, and the activation of butyrylcholinesterase that was induced by COX-2 [57]. In addition, *α*7 nAChR partial agonist (GTS21) significantly reduced LPS-induced secretion of inflammatory cytokines (TNF-*α* and IL-6), inhibited the NF-*κ*B pathway, and upregulated canonical Nrf2 antioxidant genes (HO1, TXNRD1, and GCLC) in cultured astrocytes.

### 2.5. Mitochondria

Evidences has suggested that the dysfunction of mitochondria may play an important role in the pathogenesis of MDD [58]. In depressive rodents, mitochondrial perturbation and mitochondrial component release were reported to promote cytokine generation and neuroinflammation. Meanwhile, cytokines were able to influence several mitochondrial functions, including oxidative phosphorylation and oxidative stress, and thus facilitate the neuroinflammation [58].

In microglial cells exposed to LPS, mitochondrial fission regulated mitochondrial ROS production and promoted the expression of TNF-*α*, IL-1*β*, IL-6, IL-23, iNOS, and COX-2 through the activation of NF-*κ*B and MAPK [59]. Rotenone and tebufenpyrad could induce mitochondrial impairment, enhance mitochondrial ROS generation, and amplify LPS-induced upregulation of the NLRP3 inflammasome and the generation of IL-1*β* [60]. Since the synthesis of mitochondrial DNA (mtDNA) was crucial for NLRP3 signaling in macrophages, the oxidized mitochondria may facilitate the microglia-derived inflammation [61]. The activation peripheral benzodiazepine receptor (PBR), a component of the mitochondrial permeability transition pore (PTP), significantly inhibited the LPS-induced upregulation of COX-2 and TNF-*α* [62]. Translocator protein (TSPO), an outer mitochondrial membrane protein, was upregulated in LPS-challenged microglial cells. TSPO could reverse the LPS-triggered production of the proinflammatory mediators CCL2, IL-6, and iNOS [63]. Mitochondrial uncoupling protein 2 (UCP2) knockout mice demonstrated depressive-like behaviors and lost more astrocytes in the hippocampus when exposed to chronic mild stress. UCP2 decreased ROS, negatively regulated the activation of NLRP3 inflammasome, and inhibited the production of IL-1*β* in astrocytes exposed to LPS in primary cultures [64].

### 2.6. Steroid Hormone Pathway

Steroid hormones, such as glucocorticoids (GCs) and estrogens, are well-established regulators of immune responses. Dysfunction of steroid hormones has been found in MDD. Usually, stress leads to the elevation of GCs, the subsequent activation of the hypothalamus-pituitary-adrenal (HPA) axis, and the activation of the glucocorticoid receptor (GR) in the brain, which exerts a negative feedback. However, prolonged exposure to stress leads to a defective feedback of GR [65, 66]. GR, mineralocorticoid receptor (MR), and estrogen receptor alpha (ER*α*) were all expressed in microglia. After LPS challenge, the expressions of GR, MR, and ER*α* were significantly downregulated. Corticosterone application inhibited the upregulation of TNF-*α*, IL-6, and NO induced by LPS and INF-*γ*, while 17 beta-estradiol had little effect, which suggested GR and MR were the primary steroid hormone regulators in microglial inflammatory activity [67]. Corticosterone inhibited the upregulation of excitatory amino acid transporter, GLT-1, and glutamate uptake capacity in microglia induced by LPS [68]. Chronic corticosterone exposure increased the gene expression of NLRP3 and NF-*κ*BI*α* in microglia, and even chronic corticosterone exposure potentiated the microglial proinflammatory response (TNF*α*, IL-1*β*, IL-6, and NLRP3) to LPS [69]. In microglial cells, the administration of 17 beta-estradiol (E2) or progesterone (P) dampened IL-1*β*, ASC, and NLRP3 expression after hypoxia [70]. Glucocorticoids enhanced the release of ATP from astrocytes by opening the pannexin-1 hemi-channels, which was regulated by glucocorticoid-inducible kinase-1 (SGK-1) [71]. Glucocorticoids decreased the expression of GR and AMP-activated protein kinase (AMPK) activation in cultured astrocytes. The activation of AMPK could prevent the dexamethasone-induced downregulation of GR and depression-like behavior in rats [72]. Estradiol (E2) could repress astrocyte GFAP protein expression, reorganize laminin, and enhance neurite outgrowth [73]. Corticosterone downregulated the biosynthesis of connexin43 (Cx43) but increased the degradation of Cx43 in the prefrontal cortical and hippocampus astrocyte, suggesting stress-induced dysfunction of gap junctions [74, 75].

### 2.7. Connexin in Glial Cells

Connexin (Cx)43, Cx30, Cx26, Cx40, and Cx45 are expressed in astrocytes [76, 77], while Cx43 and Cx30 are the most important Cx species contributing to gap junction channels (GJCs) or hemi-channels (HCs) [78, 79]. Energetic metabolites can be released by astrocytes through the gap-junction channels and hemi-channels that are usually used by neurons to maintain their functions [80, 81]. Also, Cx plays a role in postnatal BBB maturation [82] and neural synaptic plasticity [83]. In the gap junction protein Cx43, its carboxyl-terminal domain could modulate the function of astrocyte P2Y1 receptors [84]. *In vitro*, Cx32 and Cx36 are expressed in resting microglia, while activated microglia express Cx32, Cx36, and Cx43 [85–87]. However, *in vivo*, there were no GJCs between microglial cells and other surrounding cells [88]. Excess glutamate could be released from microglia by Cx32 hemi-channel and caused excite-neurotoxicity [89, 90]. In rat prefrontal cortices after chronic stress, Cx43 was significantly decreased, while the use of gap junction blockade in the prefrontal cortex also induced depressive-like behaviors [91]. Cx30 and Cx43 were reduced in the dorsal lateral prefrontal cortex of suicide completers [92]. Acute stress and chronic stress-induced astrocyte and microglia hemi-channel opening and caused an increase of ATP and glutamate release, which contributed to the cognitive deficits associated with major depression [93]. There were decreased gap junction channels between Cx30 of astrocyte and Cx47 of oligodendrocyte bodies and myelinated fibers in the anterior cingulate of depressed suicides [94]. Improvements in gap junction functionality concurrent with blockage of hemi-channels might be a potential target for depression treatments [95].

### 2.8. Neuropeptide in Glial Cells

Neuropeptides, such as neuropeptide Y (NPY), substance P, and galanin (GAL), were found to be affected by stress or to be involved in stress response, in some animal models or human depression patients [96]. And recently, neuropeptides were reportedly relevant to the mechanism of action of selective serotonin reuptake inhibitors [97]. NPY was found to inhibit LPS-induced NO production in N9 microglial cell line, iNOS expression, and motility through activation of NPY Y1 receptor by nuclear translocation of NF-*κ*B or inhibition of p38 activation [98, 99]. In human microglial and astrocytic cells expressing neurokinin 1 receptor (NK-1R), LPS was able to significantly increase NK-1R expression, and substance P could augment the production of inflammatory IL-6 in astrocytes [100]. GAL increased the migration of cultured microglia and upregulated class II major histocompatibility complex expression through the activation of protein kinase C [101].

### 2.9. Glial Cells, Pain, and Depression

The fact that some of the abovementioned pathways are closely involved in the pain promote us to discuss the cooccur condition of depression, pain. Pain could exacerbate the symptom of depression but the common mechanisms behind them remain to be identified [102]. Inflammation activated KYN pathway could potentially modulate neuropathic and inflammatory pain sensitivity [103]. Meanwhile, inflammasome, NF*κ*B, and NMDA receptors are known or potential target of pain therapy [104–106]. The crosstalk between pain and depression could be mediated with these signaling pathways. Further investigation of how the crosstalk is modulated may decipher the complex relationship between pain and depression.

## 3. Conclusions

**Figure 1:**
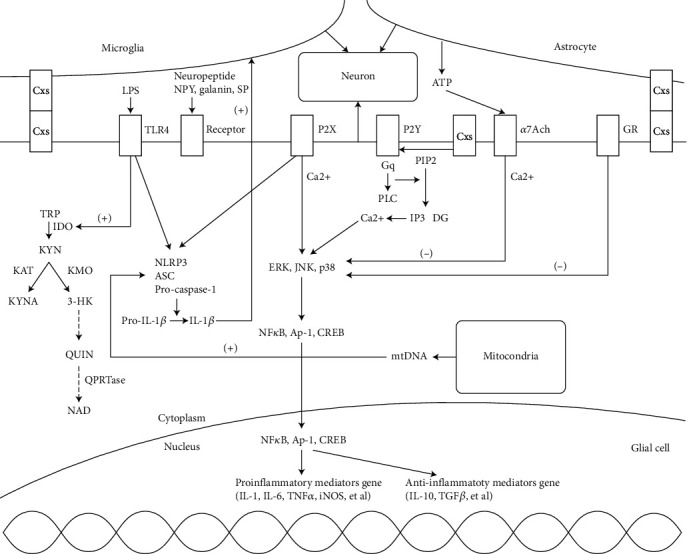
A schematic graph demonstrates the role of glial cells and the relevant signaling pathway. The surrounding microglia (upper left) and astrocyte (upper right) impact the function of neuron (middle) in multiple ways. The nuclear mechanisms of some of the pathways are graphed at the bottom.

Taken together, neuroinflammation is a very important pathological component of depression. There are many important signaling pathways woven together to form the network of astrocytes and microglia in the context of depression. Here, we summarized the recent progress on several signaling pathways, including the kynurenine pathway, the inflammasome, the purinergic pathway, the nicotinic acetylcholine pathway, mitochondria, and steroid hormone pathways, by focusing on their molecular mechanisms, respectively (Figure 1). The purpose of this summary is to update the information on the recent advances in molecular mechanisms and may provide useful information to further understand the whole picture of the interactions between neuroinflammation, glial cells, and depression (Table 1).

## Figures and Tables

**Table 1:** Summary of the pathways in depression.

Pathway	Reports	
Kynurenine	KYN pathway	The neurite branching and complexity of cortical neurons was facilitated by the IFN*γ* stimulated kynurenine pathway induction in microglia [29].
Inflammasome	MDD patients	Assembly of the NLRP3 complex in microglia activated proinflammatory response and contributed to depression [39].
Purinergic	CUMS mouse	Modulation of P2X7/NF-*κ*B pathway in microglia could attenuate the depressive-like behaviors in mice [43].
Nicotinic acetylcholine	In mouse model	Regulating alpha-7 nicotinic receptor in glial cells prevented depression-like behaviors in mice [52].
Mitochondria	UCP2 knockout mouse	Loss of mitochondrial uncoupling protein 2 (UCP2) in astrocytes aggravated depressive-like behaviors in mice [64].
Connexin	Depressed suicides	Decreased gap junction channels in the astrocytes and oligodendrocytes of the anterior cingulate of suicidal patients [94].
Neuropeptide	Neuropeptide Y	Neuropeptide Y inhibited the inflammation in microglia, which was one of key processes in depression [97].

## Data Availability

The data used to support the findings of this study are available from the corresponding author upon request.
